# Novel Method to Efficiently Create an mHealth App: Implementation of a Real-Time Electrocardiogram R Peak Detector

**DOI:** 10.2196/mhealth.8429

**Published:** 2018-05-22

**Authors:** Vadim Gliner, Joachim Behar, Yael Yaniv

**Affiliations:** ^1^ Technion-IIT Haifa Israel

**Keywords:** atrial fibrillation, arrhythmia, heart rate variability, MATLAB Mobile, mobile device

## Abstract

**Background:**

In parallel to the introduction of mobile communication devices with high computational power and internet connectivity, high-quality and low-cost health sensors have also become available. However, although the technology does exist, no clinical mobile system has been developed to monitor the R peaks from electrocardiogram recordings in real time with low false positive and low false negative detection. Implementation of a robust electrocardiogram R peak detector for various arrhythmogenic events has been hampered by the lack of an efficient design that will conserve battery power without reducing algorithm complexity or ease of implementation.

**Objective:**

Our goals in this paper are (1) to evaluate the suitability of the MATLAB Mobile platform for mHealth apps and whether it can run on any phone system, and (2) to embed in the MATLAB Mobile platform a real-time electrocardiogram R peak detector with low false positive and low false negative detection in the presence of the most frequent arrhythmia, atrial fibrillation.

**Methods:**

We implemented an innovative R peak detection algorithm that deals with motion artifacts, electrical drift, breathing oscillations, electrical spikes, and environmental noise by low-pass filtering. It also fixes the signal polarity and deals with premature beats by heuristic filtering. The algorithm was trained on the annotated non–atrial fibrillation MIT-BIH Arrhythmia Database and tested on the atrial fibrillation MIT-BIH Arrhythmia Database. Finally, the algorithm was implemented on mobile phones connected to a mobile electrocardiogram device using the MATLAB Mobile platform.

**Results:**

Our algorithm precisely detected the R peaks with a sensitivity of 99.7% and positive prediction of 99.4%. These results are superior to some state-of-the-art algorithms. The algorithm performs similarly on atrial fibrillation and non–atrial fibrillation patient data. Using MATLAB Mobile, we ran our algorithm in less than an hour on both the iOS and Android system. Our app can accurately analyze 1 minute of real-time electrocardiogram signals in less than 1 second on a mobile phone.

**Conclusions:**

Accurate real-time identification of heart rate on a beat-to-beat basis in the presence of noise and atrial fibrillation events using a mobile phone is feasible.

## Introduction

### Background

An algorithm that runs in real time and precisely calculates the heart rate from electrocardiogram (ECG) signals on a beat-to-beat basis can serve as the core of a mobile system to remotely monitor patient health [1] and issue alerts in the case of cardiac events [2,3]. Due to their increasing computational power, wireless and Bluetooth connectivity, and the ability to store data on the cloud, mobile phones and tablets can run real-time algorithms to alert the patient and communicate with the medical staff. The main challenge in designing such a mobile bundle is to develop robust, automatic algorithms that provide real-time results and can work on noisy data recorded using a portable ECG monitor, while consuming low power. Importantly, to detect potentially fatal arrhythmogenic events, very accurate detection of R peaks on the ECG is required (in addition to other waves). For example, atrial fibrillation (AF) events characterized by an irregular and often rapid heart rate [4] are currently identified retrospectively by screening the ECG signal or using other pulse signals. Because AF can lead to stroke and ventricular fibrillation, early detection of these episodes has enormous clinical impact. It has been known for a while that the R-R pattern can be used to detect AF events when they occur or even predict them [5]. The first step in identifying AF events in real time is precise, automatic detection of R peaks to calculate the heart rate on a beat-to-beat basis.

Heart rate variability (HRV) indexes can be used to detect AF events [6-8]. To precisely calculate HRV indexes, the beat interval should be identified at each heartbeat as a first step. The R peak is the dominant point in the ECG and serves as an ideal fiducial point to calculate the heart rate.

### Prior Work

Although apps to monitor heart rate using mobile device sensors do exist, they have several drawbacks. First, they work poorly on patients with heart disease versus individuals with normal heart rhythm [9]. Although a breakthrough was recently achieved in dealing with arrythmic recordings and other noise [10], it still does not provide real-time results [3]. Furthermore, only an average heart rate over a time window is provided and not the beat-to-beat interval (which is necessary for HRV analysis). Certain artifacts specific to the ECG signal can limit automated detection of the heart rate [10]: (1) sudden movement of the patient, (2) drift of the signal, (3) breathing noise, (4) wrong polarity (the ECG leads are set upside down), (5) electrical spikes from the device, (6) high frequency noise from the environment, (7) premature ventricular contraction, and (8) enlarged P or T waves. Several techniques to accurately decode the ECG have been suggested, including Fourier transform [11], Hilbert transform [11], and Wavelet transform [12], among others. These techniques require long ECG signal recordings; thus, they cannot serve as the core of a system that provides real-time R peak detection.

The recently developed MATLAB Mobile environment platform allows any algorithm, even one with high computational demands, to be converted to run on a mobile app, but its suitability for mHealth apps was never tested.

### Goal

We aim (1) to evaluate the suitability of the MATLAB Mobile platform for mHealth apps and whether it can run on any phone system, and (2) to embed in the MATLAB Mobile platform a robust real-time ECG R peak detector with low false positive and low false negative detection in the presence of AF, the most frequent arrhythmia. Because our main goal is to implement an ECG R peak detector on a mobile device, the mobile app should be compatible with any phone system.

We first present our algorithm for peak detection. The algorithm works by filtering the signal with high polynomial fit, decoding the first and second derivatives of the signals, filtering peaks that have low probability to be the R peak, and outputting the R peak location. We show that the algorithm can deal with the previously mentioned artifacts. Moreover, it can accurately identify the R peaks, as demonstrated by evaluating the algorithm’s performance on the MIT-BIH database (gold standard database from Physionet) and comparing it to other algorithms [13-15]. Later, we demonstrate how the MATLAB Mobile platform can be used to implement the algorithm on a mobile phone. Finally, we show that the algorithm can identify R peaks in real time from data acquired by a mobile ECG device.

## Methods

### Database

The proposed algorithm was tested on the MIT-BIH Arrhythmia Database [16], which includes data from patients who suffer from AF (n=25) and from healthy subjects (n=23). For each recording, the data was analyzed in its entirety regardless of whether artifacts appeared. Each record contains more than half an hour of continuous data, sampled at a rate of 360 Hz. The database is approved by an institutional review board, publicly available, and the patient information was deidentified. A total of 48 records of ECG strips from two leads (one channel) were originally obtained from 47 subjects (there are two records from the same participant) between 1975 and 1979 in Boston’s Beth Israel Hospital Arrhythmia Laboratory. The actual database contains 23 recordings of 30 minutes that were randomly chosen from a set of 4000 24-hour ambulatory ECG recordings collected from a mixed population of AF inpatients (approximately 60%) and outpatients (approximately 40%) at Boston’s Beth Israel Hospital. It also includes 25 recordings selected from the same set to include less common but clinically significant arrhythmias that are not usually present in a small random sample. The ECG recordings were made using Del Mar Avionics model 445 two-channel reel-to-reel Holter recorders. The recordings were digitized at 360 samples per second per channel with 11-bit resolution over a ±10 millivolts range and a notch filter was used to remove 60 Hz power line interference (Del Mar Avionics model 660 playback unit). Because of problems in the digitization, the analog signals from the playback unit were filtered to limit saturation in analog-to-digital conversion and for antialiasing, using a passband of 0.1 to 100 Hz relative to real time. The digitized 11-bit samples were converted into 8-bit first differences on the fly, thus limiting the slew rate to 225 millivolts per second (no major effect on the data). Two or more cardiologists independently annotated each record; disagreements were resolved and annotations for each beat (112,415 annotations overall) were included with the database.

### General Approach

**Figure 1:**
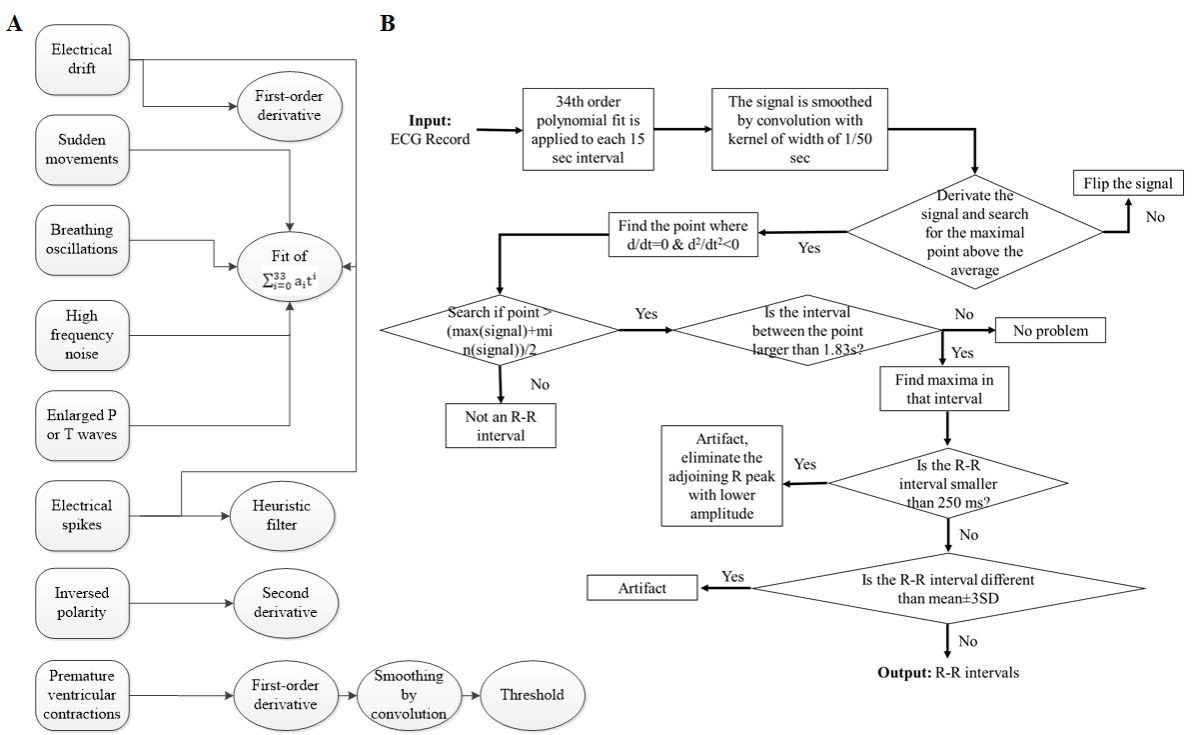
Flowchart of (A) the mathematical manipulations necessary to deal with each artifact/noise type and (B) the algorithm for identifying R peaks.

Our method for detecting the R peak includes six mathematical manipulations (Figure 1A) and nine steps (Figure 1B). The algorithm deals with sudden movement of the patients, electrical drift, breathing noise, electrical spikes, environmental high frequency noise, reverse polarity, premature ventricular contraction, and enlarged P or T waves. See Figure 1B for a step-by-step description of how the R peaks were detected. We used both physiological and data-sample criteria. To define the frequency above or below the R-R interval, we used physiological criteria (explained subsequently). However, the definition of the kernel width is based on data sampling frequency (explained subsequently).

### R Peak Detection in the Presence of Sudden Patient Movement

**Figure 2:**
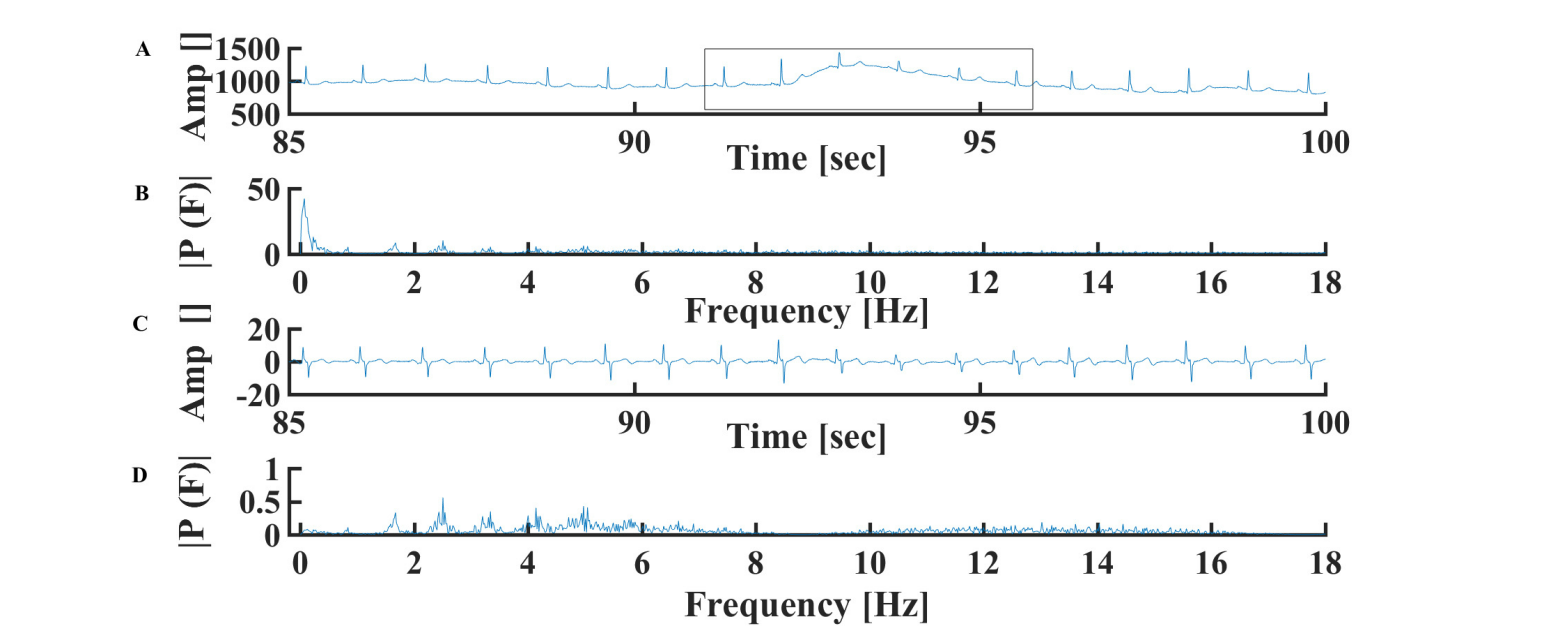
A representative example of a sudden movement artifact in the (A) time and (B) frequency of the ECG signal. Representative examples of ECG signals in (C) time and (D) frequency domains after filtering of the movement artifact. Data from MIT-BIH Arrhythmia Database #101.

**Figure 3:**
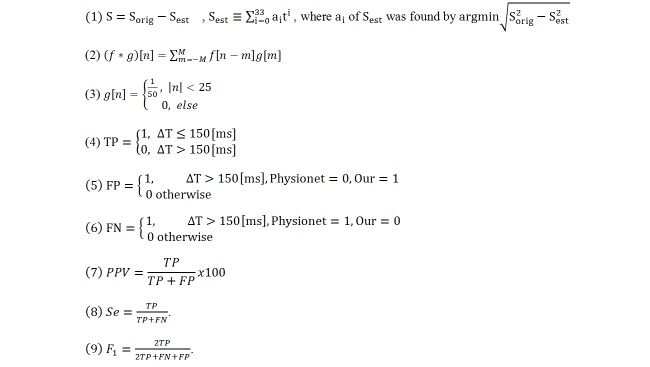
Equations. FN: false negative; FP: false positive; TN: true negative; TP: true positive; PPV:positive predictive value; Se: Sensitivity; Balanced F score (F1).

Sudden movement of the patient leads to low frequency noise in the ECG signal (Figure 2A-D). As demonstrated in Figure 2A, this type of noise increases the ECG amplitude (making it higher than the R peak itself), interfering with R peak identification. To filter this artifact and others, 34th order polynomial fit was applied to each 15-second interval (Moore-Penrose pseudoinverse), which was subtracted from the accumulated 15-second signal segment (see Equation 1 in Figure 3).

We used the data from healthy subjects to find the degree of the polynomial and we tested it on the entire database (Multimedia Appendix 1). Multimedia Appendix 1 shows the percentage of true positive (see subsequent definition) results as a function of the filter order (the entire MIT-BIH Arrhythmia Database was used). The 34th polynomial order provides the optimum results (minimal polynomial number that provides the highest true positive results) for this and other noise sources.

### R Peak Detection in the Presence of Breathing Oscillations

**Figure 4:**
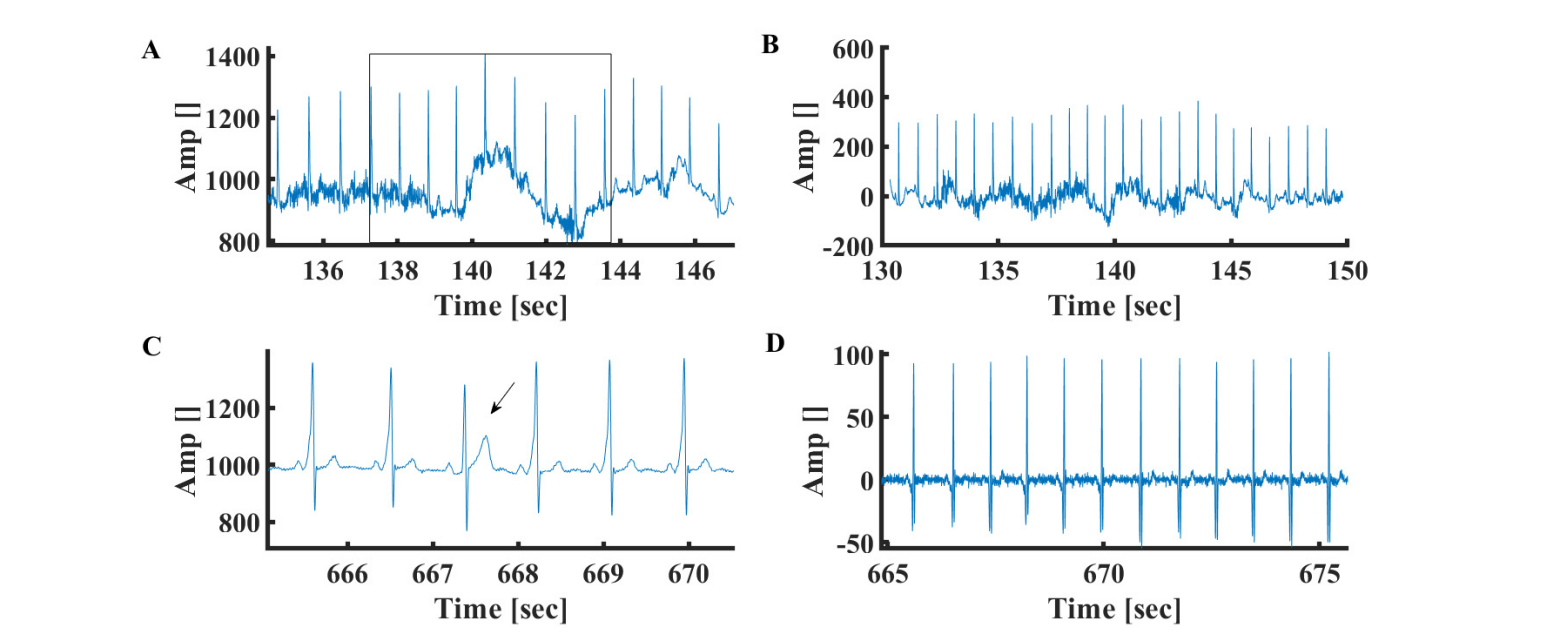
Representative example of (A) slow respiratory oscillation noise (the box represents a breathing cycle) and (B) ECG signal after respiratory oscillation filtering. Data from MIT-BIH Arrhythmia Physionet database #101. Repetitive examples of (C) enlarged T wave and (D) ECG signal after enlarged T filtering. Data from MIT-BIH Arrhythmia Database #230.

Patient breathing (due to chest movement) leads to slow fluctuation (<1 Hz) of the ECG signal (Figure 4A). To filter this artifact, a midlow polynomial fit is required. Because the signal is filtered by the high-order polynomial fit (described previously), no additional signal filtering is needed (Figure 4B).

### R Peak Detection in the Presence of High Frequency Environmental Noise

High frequency artifacts appear at 50 or 60 Hz (electrical net) or at 100 Hz (fluorescent lamps). These artifacts are filtered out by the high degree of the polynomial fit. Because the original data were notch filtered, no such examples can be illustrated.

### R Peak Detection in the Presence of Enlarged P or T Waves

As demonstrated in Figure 4C, the enlarged T wave may be detected as an R wave. To filter that noise, a middle-high polynomial fit degree must be applied (filtered by the same 34th degree polynomial fit; Figure 4D). The same steps are applied for enlarged P waves.

### R Peak Detection in the Presence of Electrical Drift

**Figure 5:**
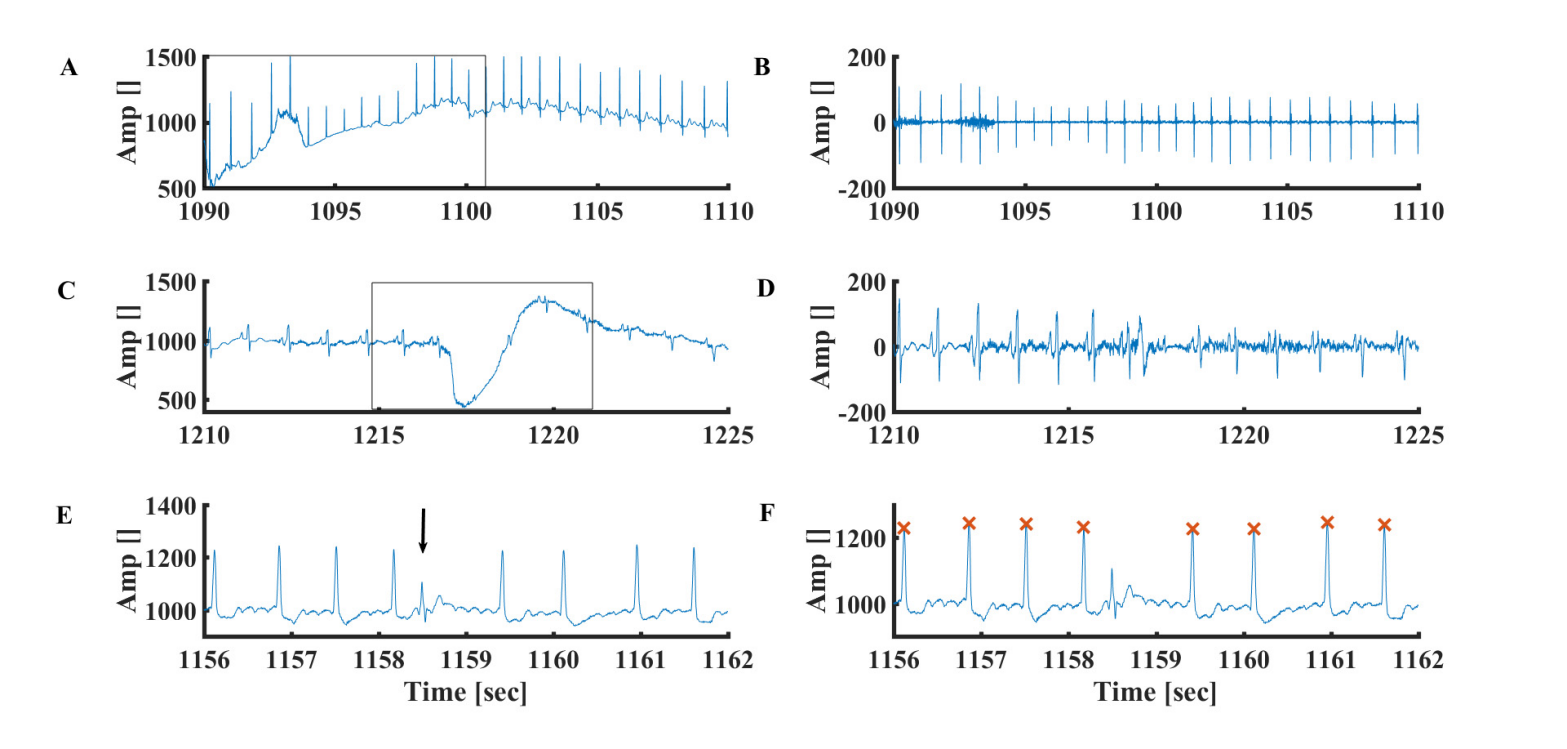
Representative example of (A) drift in the device electrical signal and (B) ECG signal after drift filtering. Data from MIT-BIH Arrhythmia Physionet database #103. Representative examples of relatively low frequency artificial electrical spikes (C) before and (D) after filtering. Data from MIT-BIH Arrhythmia Physionet database #105. Representative examples of relatively high frequency artificial electrical spikes (E) before and (F) after R peak detection. The “x” symbol represents the R peak detected by the algorithm. Data from MIT-BIH Arrhythmia Database #210.

Fluctuation in room temperature, heating of the device, or changes in the battery demand of the device (ie, power management) may lead to drift in the electrical signal. As demonstrated in Figure 5A, the drift appears as a slow and monotonic gain change of the device’s electrical signal. Because the signal is filtered by the high-order polynomial fit (described previously), no additional signal filtering is needed (Figure 5B). Note that derivation of the signal to eliminate other sources of noise also reduces low frequency drift.

### R Peak Detection in the Presence of Electrical Spikes

Random spikes often appear in the ECG signal (Figure 5C-F). These spikes are distinguished from premature beats because they are not repetitive and the consecutive R peaks are normal. Two kinds of spikes appear: (1) relatively low frequency (Figure 5C) and (2) high frequency (Figure 5E). The former artifact is filtered by the high-order polynomial fit (described previously; Figure 5D). Assuming heuristic minimal temporal distance between two adjacent R peaks, the high frequency spikes are filtered. In short, we searched for consecutive beats with temporal distance of less than 250 milliseconds (far from the maximal heart rate). In the case of adjacent peaks with distance lower than 250 milliseconds, the R peak with lower amplitude is eliminated. Such a filter is called a heuristic filter (because it is based on empirical physiological knowledge). Figure 5F demonstrates that indeed an electrical spike is not recognized as a peak.

### R Peak Detection With Reverse ECG Polarity

**Figure 6:**
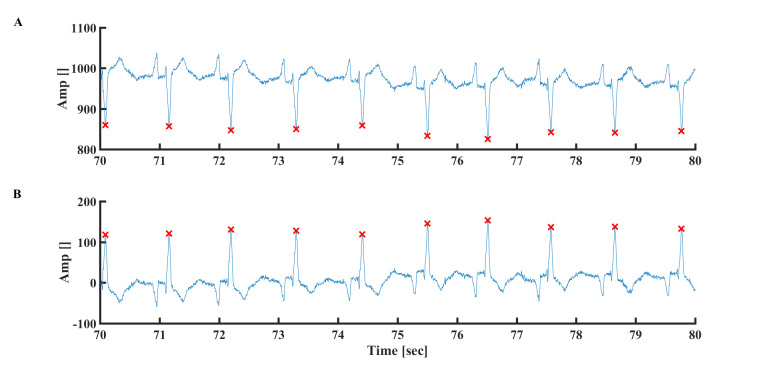
Representative examples of inverse polarity of the ECG signal (A) before and (B) after correction. Data from MIT-BIH Arrhythmia Database #108.

Swapping between ECG leads can cause negative polarity of R peaks relative to the electrical signal (Figure 6A). To overcome this problem, the signal derivative is used and maximal points are searched. If the majority of maximal points have negative values, the signal polarity is swapped (Figure 6B). In addition, the second derivative of the signal is calculated to verify that, in the case of regular polarity, the peaks are indeed maxima points, and in the case of negative polarity, the peaks are minima points.

### R Peak Detection in the Presence of Premature Contraction

**Figure 7:**
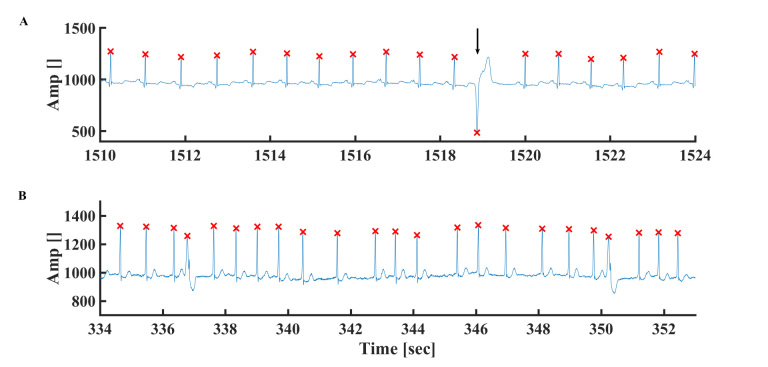
(A) Premature ventricular contraction. Data from MIT-BIH Arrhythmia Database #101. (B) Premature atrial contraction (PAC). Data from MIT-BIH Arrhythmia Database #221. The “x” symbol represents the R peak detected by the algorithm. The arrow represents PAC.

As illustrated in Figure 7A, premature ventricular contractions (PVCs) lead to early beats with similar appearance as R peaks. In the first case, the PVC polarity is positive and the algorithm detects them as R peaks; thus, no additional steps are needed (ie, the algorithm successfully finds R peaks in the presence of PVC). In the second case, premature atrial contractions (PACs) are illustrated. To overcome this problem, the signal derivative is used, and only a signal with a derivative above a certain threshold is chosen (ie, a PAC signal has a higher derivative than a regular signal). To ensure that the PAC will be detected as a peak, the ECG signal derivative was smoothed by convolution with kernel of width of 1/50 second to find the R peak (because the MIT-BIH Arrhythmia Database was sampled at 256 Hz, for this database a moving average was applied with a window width of five samples).

The convolution of two finite sequences is defined by extending the sequences to finitely supported functions on the set of integers (Equation 2 in Figure 3). When the sequences are the coefficients of two polynomials, then the coefficients of the ordinary product of the two polynomials are the convolution of the original two sequences. This is known as the Cauchy product of the coefficients of the sequences. Because we used 15-second segments, the inner product is defined (Equation 3 in Figure 3).

### Statistical Measures

Detection of R peak was performed with an assumption of causality (ie, each annotation was based on current and past R detections and not on future data). Our automatic R peak algorithm annotations were compared to the reference annotations provided by Physionet [17]. The annotations produced by the algorithm were divided into three groups as defined in Physionet [17]. If the detected R peak was in a proximity of 150 milliseconds to the reference annotations, it was identified as true positive (see Equation 4 in Figure 3). A false positive was defined if our algorithm detected a peak that did not exist in the corrected Physionet annotations in a proximity of 150 milliseconds (Equation 5 in Figure 3). Thus, false positive detection was the number of false positive events divided by the number of reference annotations.

A false negative was defined if our algorithm did not detect a peak that exists in the corrected Physionet annotations in a proximity of 150 milliseconds (Equation 6 in Figure 3). Thus, false negative detection was the number of false negative events divided by the number of reference annotations.

The positive predictive value (PPV) is defined in Equation 7 in Figure 3, sensitivity is defined in Equation 8 in Figure 3, and balanced F score is defined in Equation 9 in Figure 3.

### Other Detectors

We compared our results to three gold standard QRS detectors that show good detection in the MIT-BIH Arrhythmia Database: (1) Physionet gqrs [17], (2) Pan et al [13], and (3) Behar et al [15] (jQRS). Pan et al’s [13] QRS detector is energy based. The main operations performed by the algorithm are bandpass filter, derivative, squaring, and integration. The bandpass filter is used to reduce the influence of muscle noise, 60 Hz interference, baseline wander, and T wave interference. The signal is then differentiated to provide the QRS complex slope information, squared to make all data points positive, and a nonlinear amplification of the derivative filter is performed (which will thus emphasize the higher frequencies, contained mainly in the R wave). Next, a moving window is used to integrate the signal. Finally, an adaptive threshold is used on the integrated signal to discriminate the locations of the QRS complexes. Behar et al [15] (jQRS) used similar mathematical steps as Pan et al [13] with the following parameters: 0.6 detector threshold, 15-second window size, 150-millisecond refractory period, and 7-sample integration window.

### Mobile System

A Universal 3-12 Lead ECG Sensor (Beecardia Ltd, Haifa, Israel) was connected through a USB to a Lenovo tablet (A7-30 with CPU MTK8382-QC 1.3GHz, system memory of 1 GB RAM, and 8 GB storage capacity) with the Android 4.4 operating system. We recorded our own data, sampled at 500 Hz and uploaded to the cloud. The detector program and the acquired data were uploaded to the MATLAB Cloud. An iPhone 6s (32 GB capacity, 1.85 GHz A9 processor 64-bit architecture, 1715 mAH battery) with the iOS 10 operating system or Galaxy Note 3 (16 GB capacity, 1.3 GHz Hexa-core processor, 3200 mAh battery) with the Android 5.1.1 operating system and the MATLAB Mobile (MathWorks, Natick, MA, USA) app were used to identify the R peaks. The performance of the mobile system was compared to the performance of Lenovo Thinkpad W541 (Intel core i7pro-Quad core, clock speed 2.8 GHz processor, 16 GB RAM) with Microsoft Windows 7 Professional 64-bit edition operating system. The open-source R peak detector program can be found in Multimedia Appendix 2.

## Results

We chose the MIT-BIH Arrhythmia Database because it includes ECG strips with representative noise types and arrhythmia (see Methods) and because 40% of the ECG strips in this database include AF events (the most common arrhythmogenic events). We ran our algorithm on the entire MIT-BIH Arrhythmia Database (a total of 112,415 annotations in the 48 records). On average, our algorithm produced 0.26% false negatives and 0.58% false positives, for sensitivity of 99.7% and positive prediction of 99.4%. Note that these results were obtained after exclusion of ventricular flutter (ie, when there is no sinus rhythm at all). For statistical data, see Table 1.

Next, we compared our algorithm to some state-of-the-art QRS detectors (Pan et al [13], Physionet gqrs [17], Behar et al [15]) using the MIT-BIH Arrhythmia Database. On average, our algorithm yielded higher sensitivity, PPV, and F_1_ than the others. Note that these results were also obtained after exclusion of ventricular flutter. For statistical data, see Table 1. For statistical data of each algorithm on each record, see Multimedia Appendix 3.

To test whether the algorithm can deal with AF episodes (on which our algorithm was not trained but only its performance checked), we tested it on the AF patient data. Table 1 shows that, on average, our algorithm provided good quality results. No significant difference was found between the statistics of AF and non-AF patients. Figure 7B shows an example of how, even in the presence of AF, the algorithm can detect the R peak. Multimedia Appendix 3 shows that, on average, our algorithm’s performance in the presence of AF is superior to the others.

We then checked whether our algorithm produced different results for male and female subjects and compared the results to those of the other algorithms. We used data from 10 male and 13 female AF patients and 15 male and 10 female non-AF patients. For statistical data, see Multimedia Appendixes 4 and 5. On average, the false negative rate was higher for females than for males. Similar results were obtained for other algorithms. We also checked whether age affected the results produced by our algorithm. We used data from 17 AF patients older than 60 years, 8 AF patients younger than 60 years, 15 healthy subjects older than 60 years, and 7 healthy subjects younger than 60 years. For statistical data, see Multimedia Appendixes 6 and 7. On average, total false detections were higher in patients younger than 60 years. Similar results were obtained for other algorithms.

**Figure 8:**
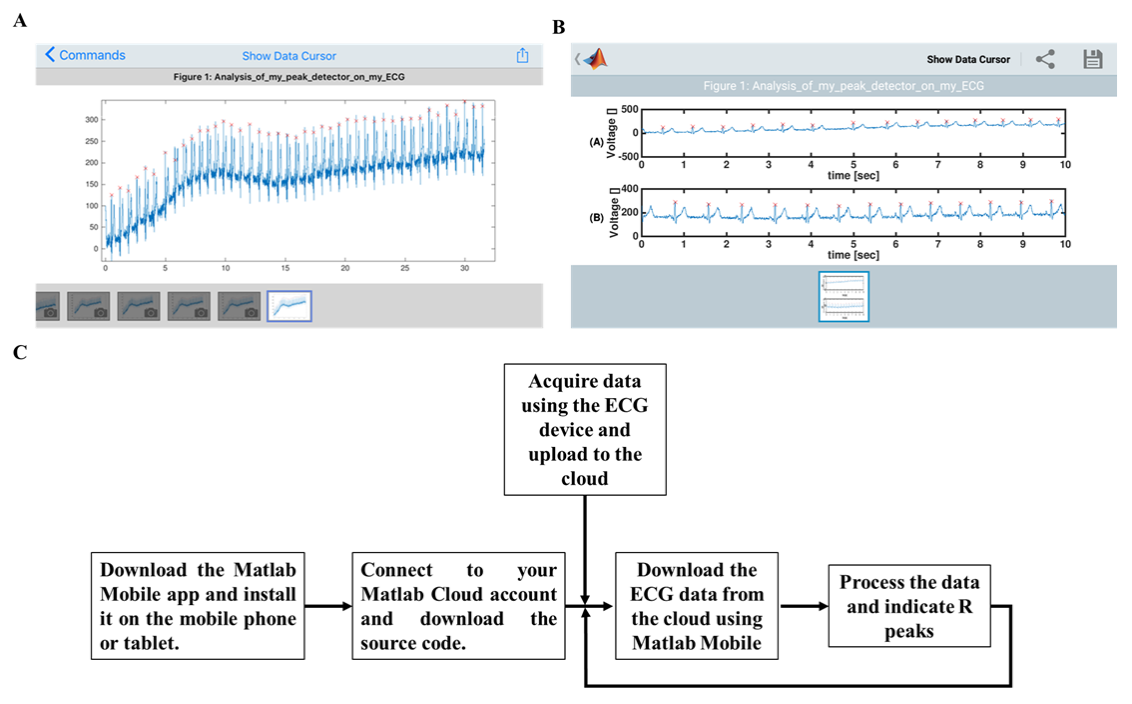
MATLAB Mobile graphic user interface with recorded data from Beecardia ECG device on (A) iOS and (B) Android systems. The “x” symbol represents R detection.

After proving the robustness of the algorithm on the “gold standard” database and proving that its performance was superior to other existing algorithms, we implemented it on a mobile phone using mobile ECG data and the MATLAB Mobile app. Figure 8A shows the MATLAB Mobile graphic user interface on both iOS and Android systems. The mobile bundle can successfully detect R peaks even in the presence of noise and drift. On average, one minute of data recording was processed by a PC (see specification in Methods) in 0.1 second and by the mobile bundle in 0.99 seconds. Figure 8B describes the steps to run the algorithm on the mobile device. Note that to compute the heart rate, the mobile device was connected to the cloud only once to download the app and once when the ECG data were download (see the flowchart in Figure 8B). Thus, it consumes low energy for communication. Indeed, 30 minutes of continuous data analysis by the app reduced the battery by only 2%.

**Table 1:** Mean statistics for the tested algorithm’s performance detecting R peaks in atrial fibrillation (AF), non-AF, and the total strips in the MIT-BIH Arrhythmia Database.

Statistic	gqrs algorithm			Pan et al algorithm			Behar et al algorithm			Our algorithm		
	AF (%)	Non-AF (%)	Total (%)	AF (%)	Non-AF (%)	Total (%)	AF (%)	Non-AF (%)	Total (%)	AF (%)	Non-AF (%)	Total (%)
False negative detection	0.4	0.1	0.3	0.7	0.5	0.3	2.1	0.5	1.2	0.4	0.3	0.3
False positive detection	0.8	0.4	0.6	0.7	0.3	0.6	5.4	1.2	3.9	0.7	0.2	0.6
Sensitivity	99.5	99.8	99.7	99.3	99.5	99.7	97.7	99.5	98.6	99.6	99.7	99.7
Positive predictive value	99.2	99.6	99.4	99.3	99.7	99.4	95.7	98.8	97.2	99.2	99.7	99.4
F_1_	99.4	99.7	99.5	99.3	99.6	99.5	96.2	99.2	97.4	99.4	99.7	99.6

## Discussion

### Principal Findings

A mobile health app with a robust R peak detector is necessary to calculate heart rate to diagnose diseases, evaluate the patient’s condition, and trigger alerts if potentially fatal events are about to occur or have just occurred. To identify real-time R peak intervals, the algorithm must deal with many kinds of common artifacts before it can be implemented on a mobile bundle. The first contribution of this paper is a new R peak detector implemented on a mobile app in approximately 2 hours of computing work. We showed that it can deal with many kinds of common artifacts, such as motion artifacts, electrical drift, breathing oscillations, electrical spikes, environmental noise, signal polarity, and premature beats. We tested its performance on a “gold standard” database that includes AF and arrhythmia events. We also showed that its performance is superior to other well-cited detection algorithms [13,15,17]. Moreover, we proved that the algorithm is robust enough to detect R peaks in real time from ECG signals recorded by a mobile device. Thus, the algorithm can be run either on either gold standard data recorded by a stationary ECG device or on other data recorded by a mobile ECG device. Most importantly, we showed that the algorithm can be run on the MATLAB Mobile platform without reducing its complexity or the ability to quickly detect R peaks.

Real-time R peak detection of healthy subjects is challenging. Performing such analysis on ECG data from patients with AF who also exhibit other arrhythmias adds a new dimension of complexity to the real-time R peak detection. Our algorithm was trained only on non-AF patients. As demonstrated in Table 1, the performance of our algorithm does not decrease in the case of AF events. Moreover, although our algorithm is only slightly better than the gqrs [17] for healthy subjects, it is superior to it for recordings with AF events.

Other state-of-the-art R peak detectors, such as that of Elgendi [18], do exist. However, such algorithms are not open source and thus it is not possible to reproduce their results. In addition, our work focuses on designing an R peak detector that can be easily used on any mobile device running MATLAB Mobile.

The second and most important contribution of this paper is an open-source code that runs on the MATLAB Mobile app, which can be used by any mobile phone. The MATLAB Mobile platform makes it possible to run the R peak detection algorithm without having to reduce its complexity. To the best of our knowledge, this is the first time that MATLAB Mobile has been used as a tool to test a mobile health app. Importantly, the MATLAB source code of our R peak detector was contributed to MATLAB Cloud, which means that any mobile device running the MATLAB Mobile app can easily download and run the code.

### Clinical Insights

Precise beat-to-beat detection of R peaks is essential for accurate HRV analysis. Even under resting conditions, ECG recordings in mammals exhibit complex beat-to-beat variations in the heartbeat intervals [19]. Although a decrease in this complexity in humans with cardiovascular diseases correlates with increased morbidity and mortality [19], an increase in HRV above a certain threshold leads to the abnormal electrical impulse propagation defined as arrhythmia (for a review see [20]). On average, AF is associated with increased HRV [20], but reduced HRV quantifying indexes are observed just before arrhythmogenic events [21]. Although the correlation between changes in heartbeat complexity and the prevalence of AF has been acknowledged for over three decades [22-24], currently there is no clinical tool that exploits this correlation to predict AF. The lack of clinical tools was largely due to the lack of a high-quality real-time automatic R peak detection algorithm. Our algorithm, and the ability to embed it on mobile device, may help to realize such a tool.

### Limitations

We cannot quantitatively test the performance of the R peak detector on the mobile device because no public annotated database exists for such devices. Nonetheless, the applicability of the algorithm is ensured by its ability to detect R peaks with low false negative and false positive detections on the gold standard database, even in the presence of all possible artifacts and types of noise. A large clinical test of the device on patients will prove its quality.

Unfortunately, because most published R peak detector algorithms are not open source and not in MATLAB language, we could not run them on MATLAB Mobile. However, because our algorithm exhibits superior performance and its running time on the MATLAB Mobile app is short, we believe that testing other algorithms on the mobile platform will not bring further insight.

### Future Work

We showed here that MATLAB Mobile is suitable to run our algorithm and find R peaks in real time. In the future we would like to use MATLAB Mobile for “hybrid” calculations: the simpler parts of the calculation will be done on a mobile phone and the more complex parts will be done in the cloud. MATLAB Mobile will switch between the two parts of the calculation.

We used AF data to demonstrate the suitability of the MATLAB Mobile platform for mHealth apps and showed that it can run on any phone system (aim 1). Thus, similar mHealth apps might also prove useful for other cardiac diseases or for diseases that require tracking of bioelectric signals from a wireless device.

### Conclusions

Our first goal in this paper was to evaluate the suitability of the MATLAB Mobile platform for mHealth apps and determine whether it can run on any phone system. We showed here that an open-source code can run on the MATLAB Mobile app and can be used to identify the R peaks. Our second goal was to embed in the MATLAB Mobile platform a robust real-time ECG R peak detector with low false positive and low false negative detection in the presence of the most frequent arrhythmia, AF. We showed here that our algorithm can deal with many kinds of common artifacts, such as motion artifacts, electrical drift, breathing oscillations, electrical spikes, environmental noise, signal polarity, and premature beats. We also showed that its performance is superior to that of other well-cited detection algorithms [13,15,17]. Moreover, we proved that the algorithm is robust enough to decode R peaks in real time from ECG signals recorded by a mobile device.

## Appendix

Multimedia Appendix 1 Percent of true positive results as a function of polynomial fit degree. The entire MIT-BIH Arrhythmia database was used. A polynomial order higher than 34 filters important information and decreases the percentage of true positive results.

Multimedia Appendix 2 Open source code.

Multimedia Appendix 3 Comparison of failed detection per recording of each algorithm run on the MIT-BIH Arrhythmia database.

Multimedia Appendix 4 Average statistics for the tested algorithm’s performance on male subjects from the MIT Arrhythmia database.

Multimedia Appendix 5 Average statistics for the tested algorithm’s performance on female subjects from the MIT Arrhythmia database.

Multimedia Appendix 6 Average statistics for the tested algorithm’s performance on patients younger than 60 in the MIT Arrhythmia database.

Multimedia Appendix 7 Average statistics for the tested algorithm’s performance on patients older than 60 in the MIT Arrhythmia database.

## Abbreviations

AF: atrial fibrillation
ECG: electrocardiogram
HRV: heart rate variability
PAC: premature atrial contraction
PVC: premature ventricular contraction
