# Supplementary figures and images for: Novel Method to Efficiently Create an mHealth App: Implementation of a Real-Time Electrocardiogram R Peak Detector
Source: JMIR Mhealth Uhealth. 2018 May 22;6(5):e118. doi: 10.2196/mhealth.8429 (PMC5989064; doi:10.2196/mhealth.8429)

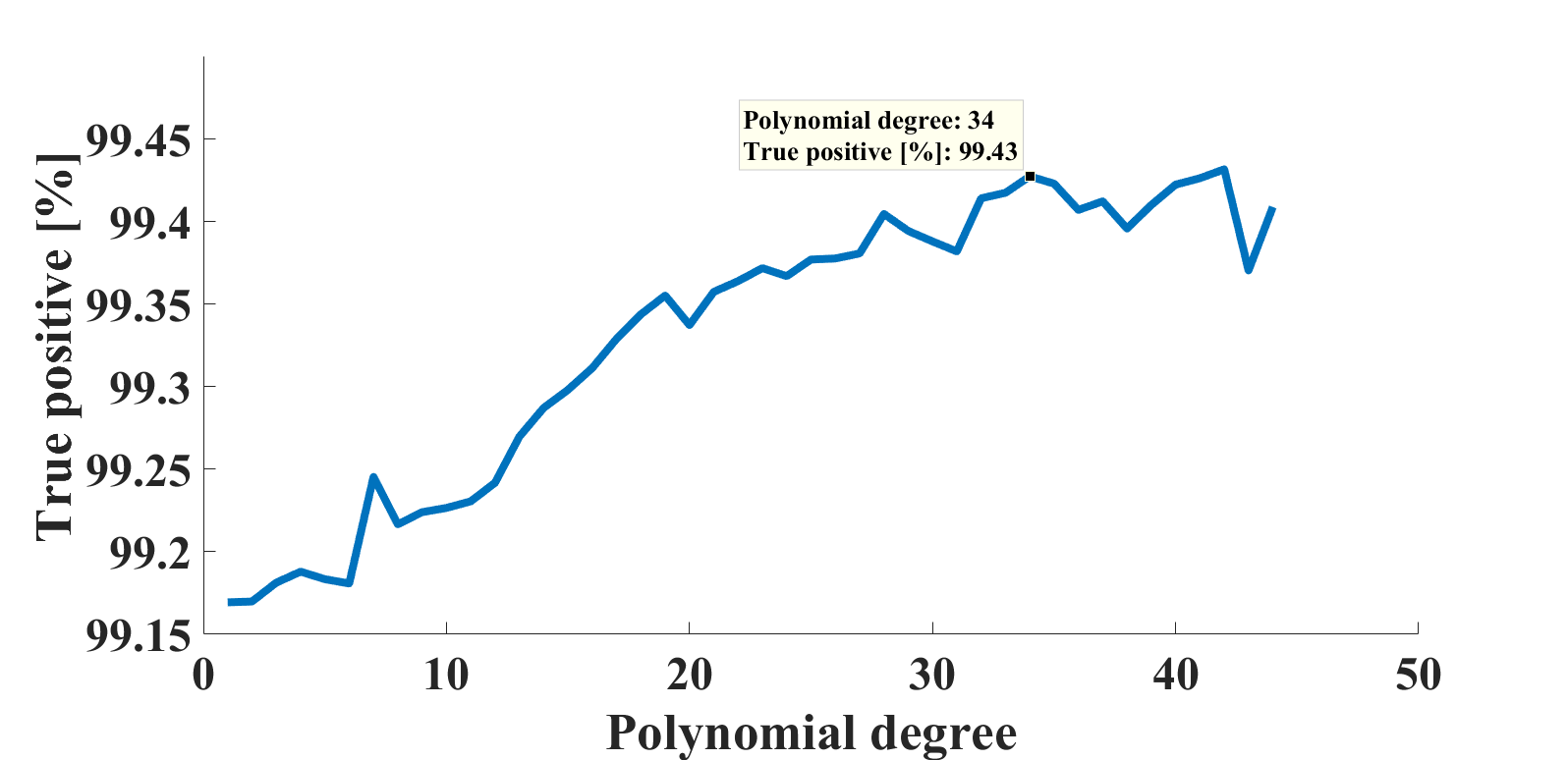

Supplement: Multimedia Appendix 1 [file mhealth_v6i5e118_app1.png]
