# Supplementary material for: Novel Method to Efficiently Create an mHealth App: Implementation of a Real-Time Electrocardiogram R Peak Detector
Source: JMIR Mhealth Uhealth. 2018 May 22;6(5):e118. doi: 10.2196/mhealth.8429 (PMC5989064; doi:10.2196/mhealth.8429)
Supplement: Multimedia Appendix 3 [file mhealth_v6i5e118_app3.pdf]

Recording number	No. of annotations	Percent of failed detection peaks from total using gqrs algorithm (%)	Percent of failed detection peaks from total using Pan et al. algorithm (%)	Percent of failed detection peaks from total using Behar et al. algorithm (%)	Percent of failed detection peaks from total using Gliner et al. algorithm (%)
100	2369	0.04	0.44	0	0.00
101	2023	0.21	0.65	0.21	0.10
102	2220	0.00	0.51	0.05	0.05
103	2359	0.05	0.43	0	0.04
104	2398	0.98	0.99	3.2	1.58
105	3026	1.61	2.23	1.2	3.60
106	2282	0.89	1.09	4.33	0.53
107	2186	0.42	0.52	1.1	0.14
108	1831	4.08	4.94	19	2.79
109	2570	0.20	0.44	0.6	0.43
111	2137	0.47	0.57	0.1	0.42
112	2944	0.04	0.47	0	0.00
113	1921	0.33	0.45	0	0.05
114	2121	1.73	0.48	0.1	0.47
115	2291	0.05	0.41	0	0.00
116	2742	0.42	0.71	3.94	0.33
117	1536	0.07	0.46	0	0.39
118	2417	0.09	0.44	0.09	0.00
119	2266	0.40	0.56	5.64	0.10
121	1914	0.27	0.49	0.42	0.05
122	2853	0.04	0.53	0	0.04
123	1748	0.13	0.66	0.2	1.58
124	1788	0.12	0.37	0	3.60
200	2853	0.38	0.66	1.15	0.53
201	2424	1.87	2.62	8.88	0.14
202	2828	0.28	0.47	0.51	2.79
203	3203	3.91	3.77	7.68	0.43
205	3193	0.41	0.83	0.5	0.42
207	2205	12.13	10.80	18.64	0.00
208	3479	1.43	1.88	26.5	0.05
209	3167	0.10	0.50	0.07	0.47
210	2968	1.88	1.76	1.55	0.00
212	3048	0.11	0.40	0	0.33
213	3326	0.18	0.65	1.7	0.39
214	2853	0.49	0.67	0.8	0.04
215	3459	0.18	0.48	0.356	0.04

217	2300	0.36	0.86	0.53	0.05
219	2574	2.92	0.61	7.98	0.04
220	2172	0.10	0.49	0	0.00
221	3027	0.25	0.62	14.36	0.00
222	3051	1.01	1.37	0.4	0.32
223	3046	1.19	1.54	6.33	0.00
228	2454	1.02	1.46	5.95	0.10
230	2554	0.09	0.58	0.04	0.05
231	2419	0.13	0.64	0.19	0.04
232	2430	0.67	0.62	56.66	1.58
233	3358	0.29	0.75	0.55	3.60
234	2779	0.25	0.62	0.18	0.53
**Average**	**2565**	**0.92**	**1.13**	**4.20**	**0.84**
