# Supplementary material for: Novel Method to Efficiently Create an mHealth App: Implementation of a Real-Time Electrocardiogram R Peak Detector
Source: JMIR Mhealth Uhealth. 2018 May 22;6(5):e118. doi: 10.2196/mhealth.8429 (PMC5989064; doi:10.2196/mhealth.8429)
Supplement: Multimedia Appendix 4 [file mhealth_v6i5e118_app4.pdf]

| | gqrs algorithm | | | Pan et al. algorithm | | | Current algorithm | | |
|---|---|---|---|---|---|---|---|---|---|
| | **AF (%)** | **Non-AF (%)** | **Total (%)** | **AF (%)** | **Non-AF (%)** | **Total (%)** | **AF (%)** | **Non-AF (%)** | **Total (%)** |
| **FND** | 0.5 | 0.05 | 0.25 | 0.8 | 0.5 | 0.6 | 0.5 | 0.05 | 0.3 |
| **FPD** | 0.5 | 0.1 | 0.3 | 0.3 | 0.03 | 0.2 | 0.7 | 0.09 | 0.4 |
| **Se** | 99.5 | 99.9 | 99.7 | 99.2 | 99.97 | 99.6 | 99.5 | 99.9 | 99.7 |
| **PPV** | 99.5 | 99.9 | 99.7 | 99.7 | 99.9 | 99.8 | 99.3 | 99.9 | 99.6 |
