# Supplementary material for: Novel Method to Efficiently Create an mHealth App: Implementation of a Real-Time Electrocardiogram R Peak Detector
Source: JMIR Mhealth Uhealth. 2018 May 22;6(5):e118. doi: 10.2196/mhealth.8429 (PMC5989064; doi:10.2196/mhealth.8429)
Supplement: Multimedia Appendix 5 [file mhealth_v6i5e118_app5.pdf]

	gqrs algorithm			Pan et al. algorithm			Current algorithm		
	**AF (%)**	**Non-AF (%)**	**Total (%)**	**AF (%)**	**Non-AF (%)**	**Total (%)**	**AF (%)**	**Non-AF (%)**	**Total (%)**
**FND**	0.4	0.2	0.3	0.6	0.5	0.6	0.2	0.15	0.17
**FPD**	1.3	0.6	0.96	1.2	0.6	0.9	0.9	0.6	0.8
**Se**	99.6	99.8	99.7	99.3	99.5	99.4	99.8	99.9	99.8
**PPV**	98.7	99.4	99.0	98.8	99.4	99.1	99.1	99.4	99.2
