# Supplementary material for: Novel Method to Efficiently Create an mHealth App: Implementation of a Real-Time Electrocardiogram R Peak Detector
Source: JMIR Mhealth Uhealth. 2018 May 22;6(5):e118. doi: 10.2196/mhealth.8429 (PMC5989064; doi:10.2196/mhealth.8429)
Supplement: Multimedia Appendix 6 [file mhealth_v6i5e118_app6.pdf]

	gqrs algorithm			Pan et al. algorithm			Current algorithm		
	**AF (%)**	**Non-AF (%)**	**Total (%)**	**AF (%)**	**Non-AF (%)**	**Total (%)**	**AF (%)**	**Non-AF (%)**	**Total (%)**
**False negative**	0.4	0.2	0.3	0.7	0.5	0.6	0.3	0.1	0.2
**False positive**	0.9	0.5	0.7	0.9	0.5	0.7	0.97	0.5	0.7
**Total False**	1.4	0.7	1.0	1.6	0.95	1.3	1.3	0.6	0.97
**Sensitivity**	99.6	99.8	99.7	99.3	99.5	99.4	99.7	99.9	99.8
**Positive prediction**	99.1	99.5	99.3	99.1	99.5	99.3	99.0	99.5	99.2
